# Ventromedial hypothalamic OGT drives adipose tissue lipolysis and curbs obesity

**DOI:** 10.1126/sciadv.abn8092

**Published:** 2022-08-31

**Authors:** Qi Wang, Bichen Zhang, Bernardo Stutz, Zhong-Wu Liu, Tamas L. Horvath, Xiaoyong Yang

**Affiliations:** ^1^ Department of Cellular and Molecular Physiology, Yale University, New Haven, CT 06520, USA.; ^2^ Department of Comparative Medicine, Yale University School of Medicine, New Haven, CT 06520, USA.; ^3^ Yale Center for Molecular and Systems Metabolism, Yale University School of Medicine, New Haven, CT 06520, USA.

## Abstract

The ventromedial hypothalamus (VMH) is known to regulate body weight and counterregulatory response. However, how VMH neurons regulate lipid metabolism and energy balance remains unknown. O-linked β-d-*N*-acetylglucosamine (O-GlcNAc) modification (O-GlcNAcylation), catalyzed by O-GlcNAc transferase (OGT), is considered a cellular sensor of nutrients and hormones. Here, we report that genetic ablation of OGT in VMH neurons inhibits neuronal excitability. Mice with VMH neuron-specific OGT deletion show rapid weight gain, increased adiposity, and reduced energy expenditure, without significant changes in food intake or physical activity. The obesity phenotype is associated with adipocyte hypertrophy and reduced lipolysis of white adipose tissues. In addition, OGT deletion in VMH neurons down-regulates the sympathetic activity and impairs the sympathetic innervation of white adipose tissues. These findings identify OGT in the VMH as a homeostatic set point that controls body weight and underscore the importance of the VMH in regulating lipid metabolism through white adipose tissue–specific innervation.

## Teaser

The VMH regulates lipid metabolism through white adipose tissue innervation, and OGT in VMH neurons plays an antiobesogenic role.

## INTRODUCTION

Metabolic disorders are a growing public health concern. The dysregulation of the central nervous system (CNS) control of metabolism is implicated in various metabolic disorders, including obesity and diabetes (*1*–*5*), in which the hypothalamus plays an essential role (*6*). Among various hypothalamic nuclei, the ventromedial hypothalamus (VMH) has been known to regulate body weight, food intake, and glucose homeostasis (*7*, *8*). Optogenetic, chemogenetic, and electromagnetic manipulations of the neuronal activity of VMH neurons have provided direct evidence for the role of the VMH in regulating feeding, counterregulatory response, and emotional states (*9*–*11*). Previous studies have also suggested that VMH neurons sense metabolic signals and coordinate with peripheral tissues to regulate whole-body energy balance and glucose homeostasis (*12*–*16*). However, the underlying mechanisms by which VMH neurons respond to metabolic challenges to maintain metabolic homeostasis remain largely unknown.

O-linked β-d-*N*-acetylglucosamine (O-GlcNAc) modification (O-GlcNAcylation) is a dynamic and reversible posttranslational modification that regulates a wide span of cellular activities including transcription, epigenetic programs, and cell signaling (*17*–*20*). Maintenance of O-GlcNAc homeostasis is essential for optimal cellular function and metabolic homeostasis, while disruption of O-GlcNAc homeostasis is implicated in the pathogenesis of various diseases including diabetes and neurodegeneration (*18*). O-GlcNAcylation is controlled by one pair of enzymes, with O-GlcNAc transferase (OGT) catalyzing its addition and O-GlcNAcase catalyzing its hydrolytic removal (*21*, *22*). In peripheral tissues, OGT has been shown to mediate nutritional or hormonal regulation of tissue physiology to maintain whole-body metabolic homeostasis (*23*–*31*). In contrast to a large body of evidence that has revealed the specific role of OGT in peripheral tissues, the role of central OGT and O-GlcNAcylation in different brain regions remains largely unknown. Previous studies have shown that OGT responds to nutritional and hormonal signals in a brain region–specific manner and regulates distinct aspects of metabolism (*32*–*35*). Despite these findings, however, whether and how ventromedial hypothalamic OGT regulates lipid metabolism and whole-body energy balance remains elusive.

Here, we report that ventromedial hypothalamic O-GlcNAcylation increases in response to fasting. Genetic ablation of OGT in the VMH leads to obesity and reduced energy expenditure in mice fed a normal chow diet. The obesity phenotype is associated with adipocyte hypertrophy and reduced lipolysis of white adipose tissues mediated by the protein kinase A (PKA)–hormone-sensitive lipase (HSL) signaling pathway. Ablation of OGT also inhibits the neuronal activity of VMH neurons, reduces sympathetic activity, and impairs the sympathetic innervation of white adipose tissues. These findings reveal that OGT in the VMH is required for promoting lipid catabolism and maintaining energy balance by regulating the VMH neuronal activity and the sympathetic outflow to white adipose tissues. This study establishes the function of the VMH in regulating lipid metabolism through white adipose tissue–specific innervation. We further unravel an essential role for OGT in regulating VMH neuronal activity and constraining body weight gain.

## RESULTS

### Fasting increases OGT expression and O-GlcNAcylation level in the VMH

**Fig. 1.**
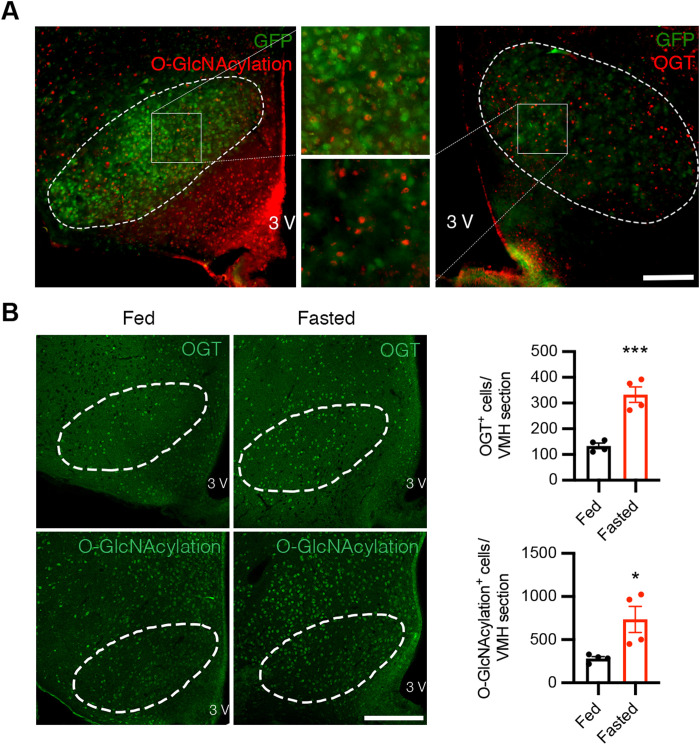
Fasting increases OGT expression and O-GlcNAcylation level in the VMH. (**A**) Immunostaining of O-GlcNAcylation (left; red) and OGT (right; red) of green fluorescent protein (GFP)–labeled SF1 neurons (green) in the VMH showing colocalization of O-GlcNAcylation/OGT and SF1 in the VMH. Scale bar, 200 μm. (**B**) Representative immunostaining of OGT (top; green) and O-GlcNAcylation (bottom; green) in the VMH of mice fed ad libitum or fasted for 24 hours. Quantifications of the average OGT/O-GlcNAcylation–positive cells in the VMH are shown on the right. Scale bar, 200 μm. Four mice each for the fed or fasted group were included in the quantifications. Data are shown as means ± SEM. *P < 0.05 and ***P < 0.001 by unpaired Student’s *t* test.

O-GlcNAcylation and OGT are abundant under basal conditions in various brain regions and cell types. Immunofluorescent staining demonstrated that subsets of VMH neurons have relatively high levels of OGT expression and O-GlcNAcylation in mice fed a normal chow diet ad libitum (Fig. 1A). To understand whether VMH O-GlcNAcylation responds to nutritional cues, we challenged the mice with fasting and found that food deprivation for 24 hours significantly increased O-GlcNAcylation level and OGT expression in the VMH (Fig. 1B). This suggests that O-GlcNAcylation level and OGT expression in the VMH fluctuate in response to changes in nutrient levels and energy demands, which also reveals the possibility that OGT in the VMH serves as a stress and metabolic sensor to regulate whole-body metabolic homeostasis.

### Loss of OGT in SF1 neurons increases body weight and adiposity

**Fig. 2.**
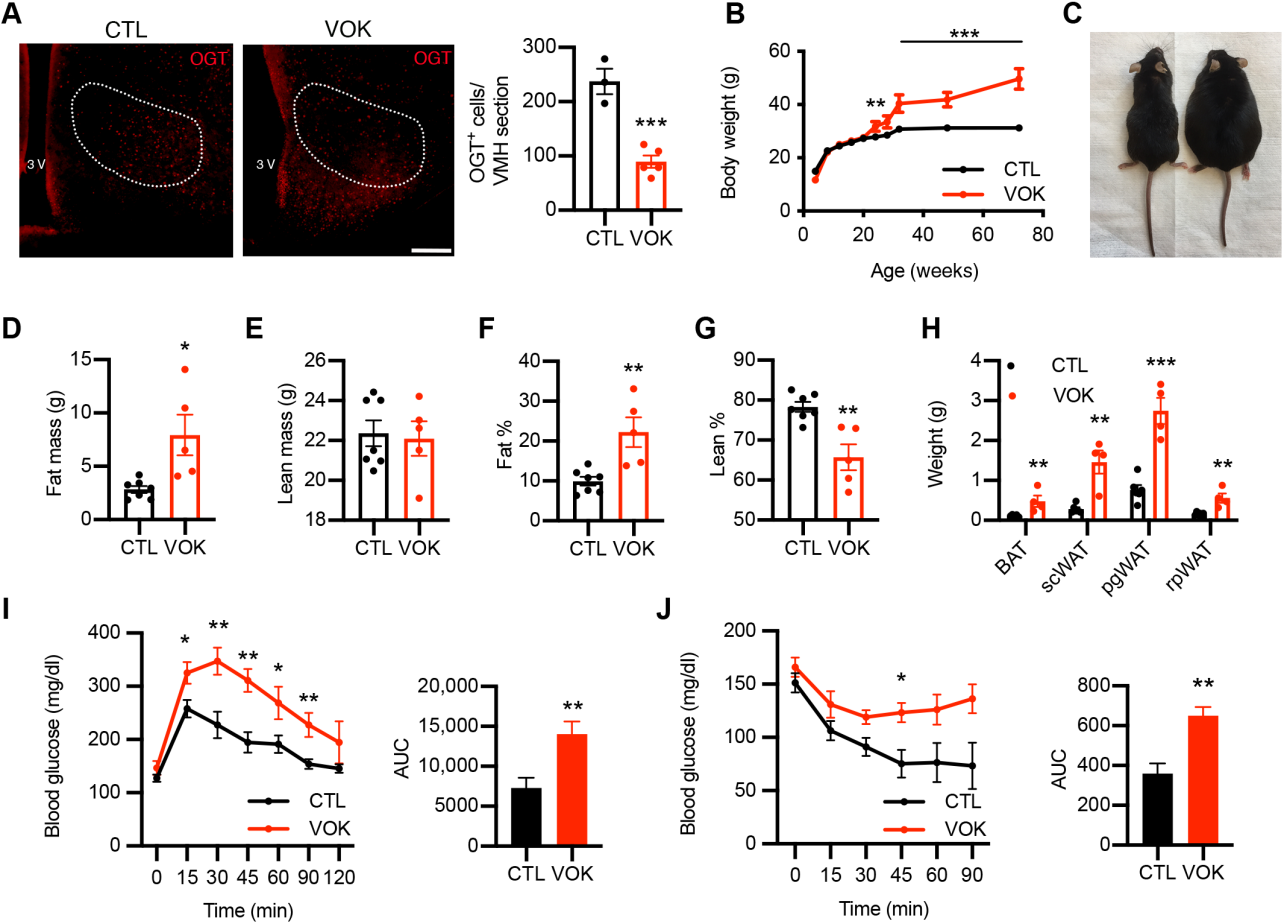
Chronic deletion of OGT in SF1 neurons leads to obesity in mice fed a normal chow diet. (**A**) Immunostaining of OGT in the VMH of CTL and VOK mice. Quantifications of OGT-positive cells in the VMH are shown on the right. Scale bar, 200 μm. (**B**) Body weight monitoring of male CTL and VOK mice fed a normal chow diet. (**C**) Representative image of CTL and VOK mice at 18 months of age. (**D**) Fat mass of male CTL and VOK mice at 6 months of age. (**E**) Lean mass of male CTL and VOK mice at 6 months of age. (**F**) Percentage of fat mass to body weight of male CTL and VOK mice at 6 months of age. (**G**) Percentage of lean mass to body weight of male CTL and VOK mice at 6 months of age. (**H**) Weight of different adipose tissues of male CTL and VOK mice at 18 months of age. (**I**) Glucose tolerance test of male CTL and VOK at 18 months of age. (**J**) Insulin tolerance test of male CTL and VOK mice at 18 months of age. CTL: *n* = 6 to 10; VOK: *n* = 4 to 8. Data are shown as means ± SEM. *P < 0.05, **P < 0.01, and ***P < 0.001 by unpaired Student’s *t* test. AUC, area under the curve.

Steroidogenic factor 1 (SF1) is considered the marker for VMH neurons in the CNS because of its specific and abundant expression in this region of the brain (*7*, *36*). To understand how changes in VMH O-GlcNAcylation affect metabolism, we generated SF1 neuron-specific OGT knockout (VOK) mice and control littermates (CTL) by crossing *Ogt*-floxed mice with *Sf1-Cre* mice. VOK mice showed reduced OGT expression in the VMH, confirming deletion efficiency (Fig. 2A). We also confirmed that deletion of OGT in SF1 neurons did not affect the gross cytoarchitecture or neuron numbers of the VMH (fig. S1A). The body weight of male VOK and CTL mice was monitored, and we found that, when fed a normal chow diet, VOK mice showed significantly higher body weight compared to CTL mice starting at the age of 24 weeks (Fig. 2, B and C). Along with obesity, VOK mice also showed increased adiposity, with higher fat mass and comparable lean mass relative to CTL mice (Fig. 2, D to G). In addition, VOK mice had increased masses of various adipose tissues, including brown adipose tissue (BAT), subcutaneous white adipose tissue (scWAT), perigonadal white adipose tissue (pgWAT), and retroperitoneal white adipose tissue (rpWAT) (Fig. 2H). In association with high adiposity, VOK mice showed glucose intolerance and insulin resistance (Fig. 2, I and J). Together, these data show that loss of OGT in SF1 neurons led to increased adiposity and obesity in mice.

### Loss of OGT in SF1 neurons reduces energy expenditure and lipolytic activity

**Fig. 3.**
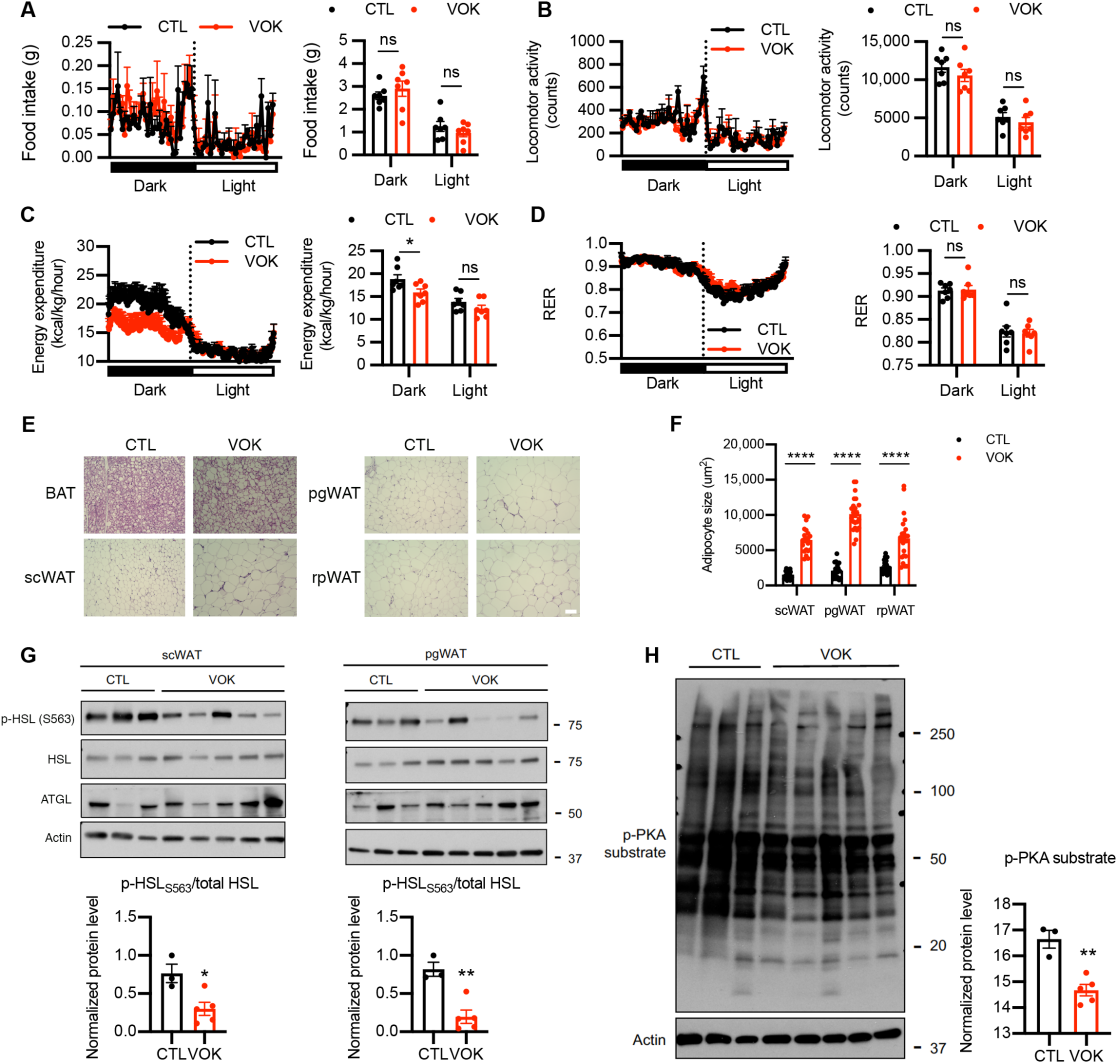
Mice with SF1 neuron-specific OGT deletion show decreased energy expenditure and reduced lipolytic activity of white adipose tissues. (**A** to **D**) Metabolic cage analysis of food intake (A), physical activity (B), energy expenditure (C), and RER (D) of CTL and VOK mice. (**E**) Hematoxylin and eosin (H&E) staining of BAT, scWAT, pgWAT, and rpWAT of CTL and VOK mice after 6 hours of fasting. Scale bar, 50 μm. (**F**) Adipocyte sizes in different white adipose tissues of CTL and VOK mice. Individual dots represent individual adipocytes from three CTL and three VOK mice. (**G**) Western blot of p-HSL_S563_, HSL, and adipose triglyceride lipase (ATGL) in scWAT and pgWAT of CTL and VOK mice after 6 hours of fasting. Densitometric quantifications for the ratio of p-HSL_S563_ to total HSL are shown below. (**H**) Western blot of p-PKA substrate in scWAT of CTL and VOK mice after 6 hours of fasting. Densitometric quantification is shown on the right. CTL: *n* = 7 and VOK: *n* = 7 for the quantifications of the metabolic cage analysis. CTL: *n* = 3 and VOK: *n* = 5 for the representative traces of the metabolic cage analysis. Mice were aged 19 to 20 weeks before the body weight divergence. Data are shown as means ± SEM. *P < 0.05, **P < 0.01, and ****P < 0.0001 by unpaired Student’s *t* test. ns, not significant.

To determine potential reasons contributing to the obesity of VOK mice, body weight–matched VOK and CTL littermates were maintained on a normal chow diet and subjected to metabolic cage studies. Although food intake and physical activity of VOK mice were comparable to those of CTL littermates (Fig. 3, A and B), VOK mice showed significantly reduced energy expenditure during dark cycles than CTL littermates (Fig. 3C). VOK mice also showed comparable respiratory exchange ratio (RER) to CTL littermates, suggesting that substrate utilization was not altered by loss of OGT in SF1 neurons (Fig. 3D).

As VOK mice showed increased adipose tissue masses (Fig. 2H), we further investigated whether they showed any maladapted metabolic function in their white adipose tissues, which contributes to their obesity (*37*) and higher adiposity. Hematoxylin and eosin (H&E) staining suggested that VOK mice had increased lipid accumulation in their BAT (Fig. 3E). They also exhibited adipocyte hypertrophy in scWAT, pgWAT, and rpWAT (Fig. 3, E and F). To determine whether the lipolytic activity was altered in white adipose tissues of VOK mice, we investigated the changes in the activity of the canonical lipolytic pathway of adipocytes. Western blot results showed that the protein level of phosphorylated HSL (phospho-HSL) at its stimulatory site S563 was down-regulated in scWAT and pgWAT of VOK mice (Fig. 3G). PKA phosphorylates HSL, promotes HSL translocation from cytosol to lipid droplets, and increases its hydrolyase activity (*37*, *38*). The PKA activity was also down-regulated in VOK mice, suggested by an overall lower level of phospho-PKA substrates in scWAT (Fig. 3H). These results show that the canonical lipolytic pathway was down-regulated in white adipose tissues of VOK mice, which explains increased adiposity and obesity observed in these mice.

### Loss of OGT in SF1 neurons impairs counterregulatory response, decreases sympathetic activity, and reduces sympathetic innervation of white adipose tissues

**Fig. 4.**
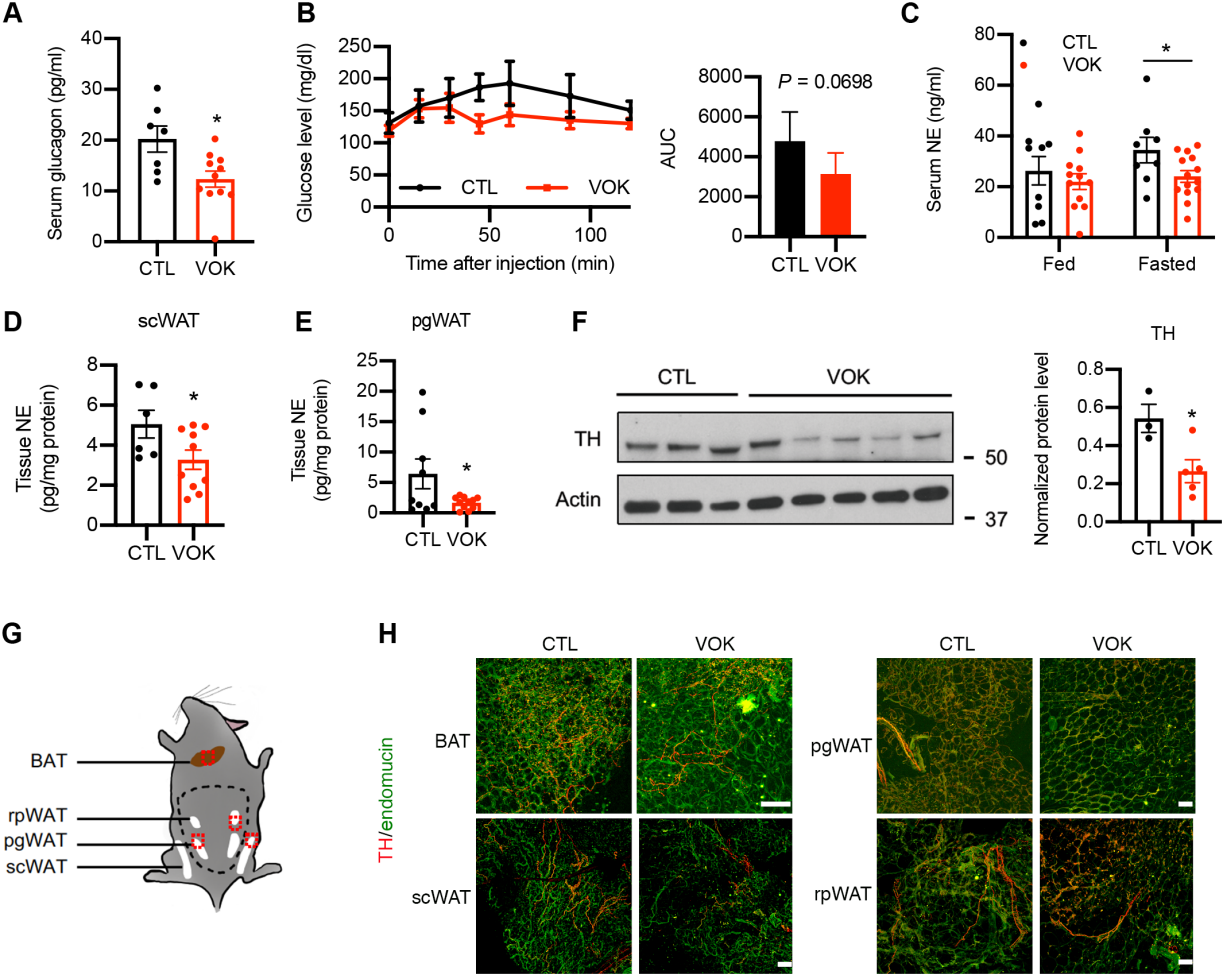
Mice with SF1 neuron-specific OGT deletion show reduced activity of the sympathetic nervous system and impaired sympathetic innervation of scWAT. (**A**) Serum glucagon levels of CTL and VOK mice after 15 hours of fasting. (**B**) Blood glucose levels of CTL and VOK mice after 2DG challenge. Quantification is shown on the right. (**C**) Serum norepinephrine levels of CTL and VOK mice fed ad libitum and after 15 hours of fasting. (**D** and **E**) Tissue norepinephrine levels in scWAT (D) and pgWAT (E) of CTL and VOK mice after 6 hours of fasting. (**F**) Western blot of tyrosine hydroxylase (TH) in scWAT of CTL and VOK mice after 6 hours of fasting. Densitometric quantification is shown on the right. (**G**) Workflow showing the process of tissue immunostaining of TH in adipose tissues of CTL and VOK mice at 7 months of age. Red dotted squares indicate the 2-mm^3^ tissue cubes used for immunostaining. (**H**) Representative immunostaining of TH (red) and endomucin (green) in BAT, scWAT, pgWAT, and rpWAT of CTL and VOK mice fed ad libitum. Scale bars, 100 μm. CTL: *n* = 7 to 9 and VOK: *n* = 10 to 15 for glucagon and norepinephrine enzyme-linked immunosorbent assay. CTL: *n* = 4 and VOK: *n* = 5 for the 2DG challenge. CTL: *n* = 3 and VOK: *n* = 3 for tissue immunostaining. Mice were fed a normal chow diet. The experiments were performed on CTL and VOK mice aging 14 to 20 weeks before body weight divergence. Data are shown as means ± SEM. *P < 0.05 by unpaired Student’s *t* test.

To cope with fasting, it is critical to maintain the blood glucose level within an appropriate physiological range. The VMH has been shown to initiate counterregulatory response to glucopenia or hypoglycemia (*13*–*15*), and we have shown that OGT expression in the VMH was increased after fasting (Fig. 1B). We then sought to determine whether loss of OGT in SF1 neurons affects counterregulatory response. By assaying the circulating glucagon levels of VOK and CTL mice after 15 hours of fasting, we found that VOK mice showed lower serum glucagon levels compared to CTL littermates (Fig. 4A). In agreement with this, after the administration of 2-deoxyglucose (2DG) to mimic glucose deprivation, VOK mice exhibited a trend of impaired counterregulatory response (Fig. 4B). These results show that OGT in SF1 neurons is necessary to maintain counterregulatory response.

In addition to counterregulatory response, fat mobilization of white adipose tissues through lipolysis is initiated during fasting to sustain energy supply. On the basis of our observation that OGT deletion in SF1 neurons down-regulated the lipolytic activity of white adipose tissues (Fig. 2, G and H), we sought to determine how OGT deletion in SF1 neurons affected adipose tissue lipolysis. We found that, although VOK and CTL mice showed comparable serum levels of norepinephrine when fed ad libitum, the norepinephrine levels in VOK mice were significantly lower compared to CTL littermates after 15 hours of fasting (Fig. 4C), suggesting that VOK mice had a reduced activity of the sympathetic nervous system. In addition, VOK mice demonstrated reduced local sympathetic activities in their scWAT and pgWAT, as indicated by lower tissue norepinephrine levels and tyrosine hydroxylase expression in white adipose tissues compared to CTL littermates (Fig. 4, D to F).

Sympathetic nerves have been shown to exhibit neuroplasticity in terms of remodeling sympathetic innervation of organs (*39*–*41*). To determine whether loss of OGT in SF1 neurons affects sympathetic innervation of adipose tissues, adipose tissues were collected and costained with tyrosine hydroxylase and endomucin (Fig. 4G). After tissue clearance, the structure and density of sympathetic nerve fibers and blood vessels were visualized. We found that VOK mice showed scattered sympathetic innervation and reduced sympathetic nerve density in their scWAT, pgWAT, and rpWAT compared to CTL littermates. In contrast, the sympathetic innervation of BAT was comparable between CTL and VOK littermates (Fig. 4H). These results show that OGT in VMH neurons is required for maintaining sympathetic activity and sympathetic innervation of white adipose tissues, which underpins the reduced lipolytic activity in white adipose tissues of VOK mice.

### Postnatal deletion of OGT in VMH neurons leads to obesity, reduced energy expenditure, and impaired WAT lipolysis associated with down-regulated sympathetic activity and innervation

**Fig. 5.**
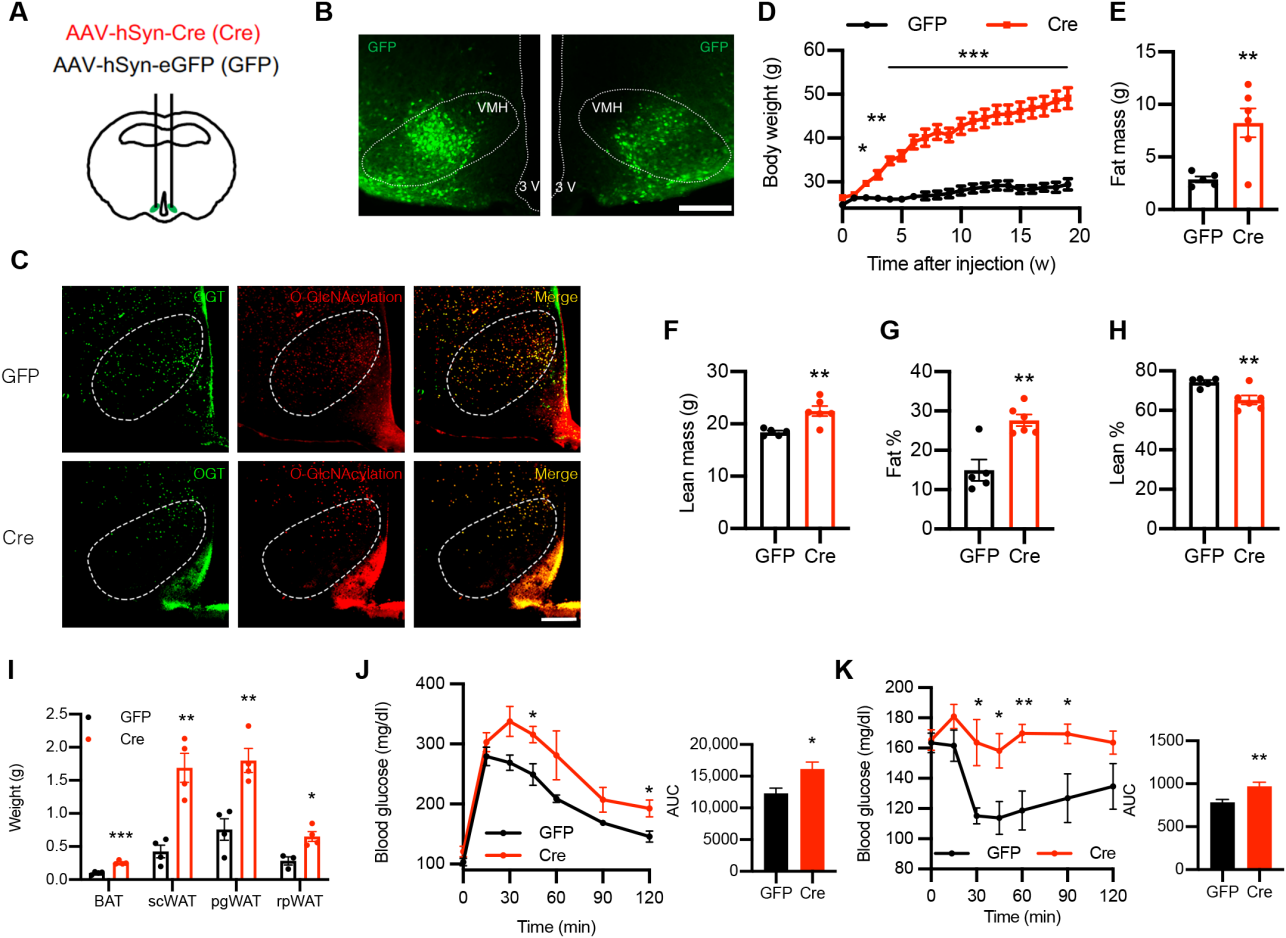
Acute deletion of OGT in VMH neurons leads to obesity in mice fed a normal chow diet. (**A**) Schematic showing the generation of control (GFP) and VMH neuron-specific OGT knockout (Cre) mice by stereotaxically injecting AAV-GFP or AAV-Cre bilaterally into the VMH of male *Ogt^flox^* mice at 12 weeks of age. (**B**) Confirmation of the AAV injection coordinate. Scale bar, 200 μm. (**C**) Validation of OGT deletion and reduction of O-GlcNAcylation level in the VMH of Cre mice. Scale bar, 200 μm. OGT was imaged using Alexa Fluor 647 with the green pseudo-color. (**D**) Body weight monitoring of GFP and Cre mice fed a normal chow diet. (**E**) Fat mass of GFP and Cre mice 4 weeks after stereotaxic virus injection. (**F**) Lean mass of GFP and Cre mice 4 weeks after stereotaxic virus injection. (**G**) Percentage of fat mass to body weight of GFP and Cre mice 4 weeks after stereotaxic virus injection. (**H**) Percentage of lean mass to body weight of GFP and Cre mice 4 weeks after stereotaxic virus injection. (**I**) Weight of different adipose tissues of GFP and Cre mice 32 weeks after stereotaxic virus injection. (**J**) Glucose tolerance test of GFP and Cre mice 5 weeks after stereotaxic virus injection. (**K**) Insulin tolerance test of GFP and Cre mice 6 weeks after stereotaxic virus injection. GFP: *n* = 8 and Cre: *n* = 8 for body weight monitoring. GFP: *n* = 4 and Cre: *n* = 4 for body composition measurements, weights of adipose tissues, glucose, and insulin tolerance test. Data are shown as means ± SEM. *P < 0.05, **P < 0.01, and ***P < 0.001 by unpaired Student’s *t* test.

To further verify that the aforementioned phenotypes in VOK mice are attributed to OGT deletion in VMH neurons, but not the effects of developmental compensation or OGT deletion in peripheral SF1-expressing tissues (*42*), adeno-associated virus–enhanced green fluorescent protein (AAV-eGFP) (GFP) or AAV-Cre (Cre) was bilaterally injected into the VMH of 12-week-old male *Ogt*-floxed mice to achieve postnatal and acute deletion of OGT in VMH neurons (Fig. 5, A and B). Bilateral injection of the Cre virus induced VMH neuron-specific deletion of OGT, followed by decreased O-GlcNAcylation level in the VMH (Fig. 5C). We found that Cre virus–injected mice recapitulated the obesity phenotype of VOK mice when fed a normal chow diet, but much more rapidly (Fig. 5, D to H, and fig. S1C). In addition, Cre virus–injected mice showed increased masses in different adipose tissues (Fig. 5I). They also showed insulin resistance and reduced glucose tolerance compared to GFP virus–injected mice associated with their body weight gain (Fig. 5, J and K). These data further confirm that OGT in VMH neurons is necessary to maintain whole-body energy balance, and loss of OGT in VMH neurons leads to rapid weight gain and increased adiposity.

**Fig. 6.**
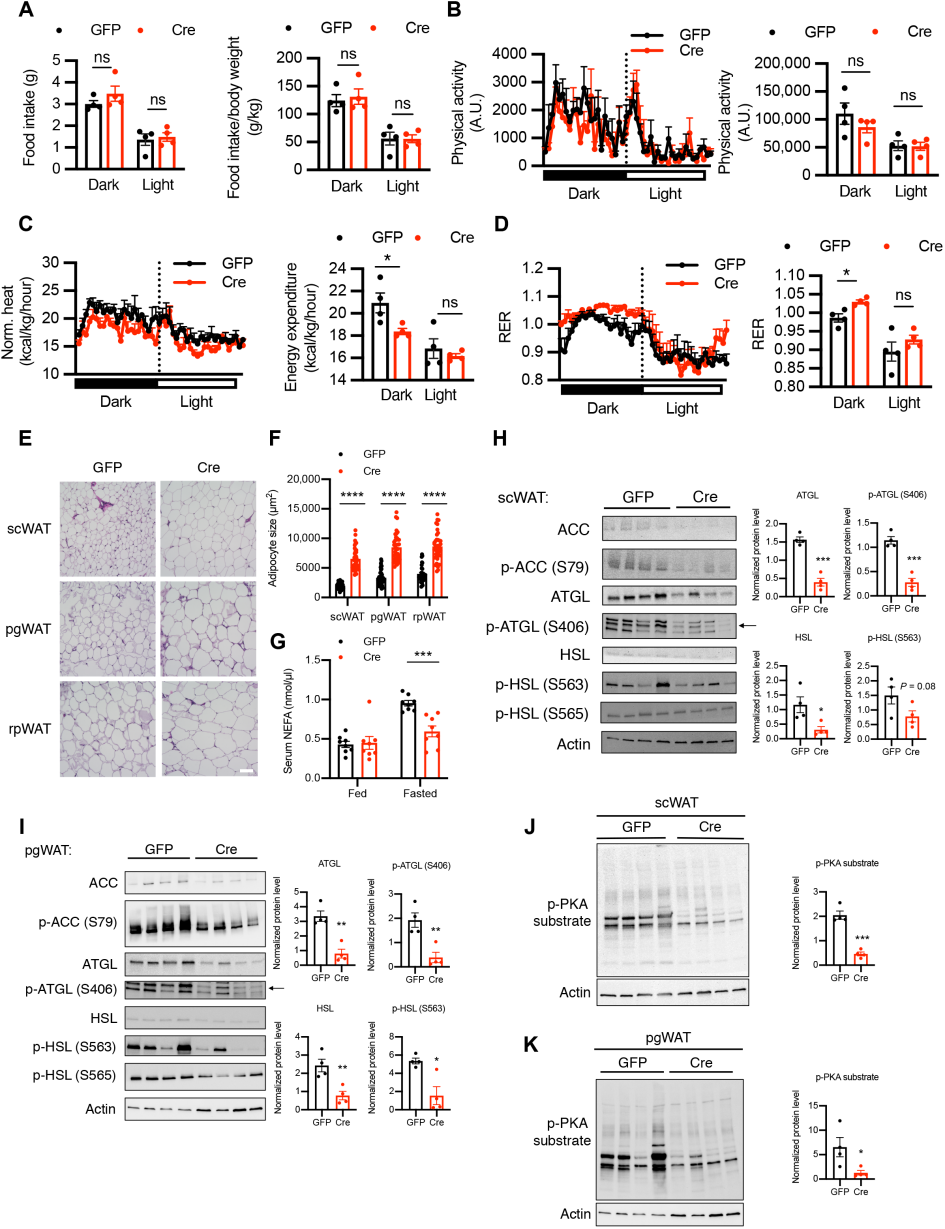
Mice with acute deletion of OGT in VMH neurons show reduced energy expenditure and adipocyte hypertrophy associated with reduced lipolysis of white adipose tissues. (**A** to **D**) Metabolic cage analysis of food intake (A), physical activity (B), energy expenditure (C), and RER (D) of GFP and Cre mice 2 weeks after stereotaxic virus injection before body weight divergence. (**E**) H&E staining of scWAT, pgWAT, and rpWAT of CTL and VOK mice after 6 hours of fasting. Scale bar, 100 μm. (**F**) Adipocyte sizes in different white adipose tissues of CTL and VOK mice. Individual dots represent individual adipocytes from three GFP and four Cre mice. (**G**) Serum nonesterified fatty acid (NEFA) levels of GFP and Cre mice fed ad libitum and after 15 hours of fasting. (**H** and **I**) Western blot of acetyl-CoA carboxylase (ACC), p-ACC (S79), ATGL, p-ATGL (S406), HSL, p-HSL (S563), and p-HSL (S565) in scWAT (H) and pgWAT (I) of GFP and Cre mice after 6 hours of fasting. Densitometric quantifications for the expression levels of ATGL, p-ATGL (S406), HSL, and p-HSL (S563) are shown on the right. (**J** and **K**) Western blot of p-PKA substrate in scWAT (J) and pgWAT (K) of GFP and Cre mice after 6 hours of fasting. Densitometric quantification is shown below. GFP: *n* = 4 and Cre: *n* = 4 for metabolic cage analysis. GFP: *n* = 8 to 10 and Cre: *n* = 8 to 10 for serum NEFA. Data are shown as means ± SEM. *P < 0.05, **P < 0.01, ***P < 0.001, and ****P < 0.0001 by unpaired Student’s *t* test. A.U., arbitrary units.

In addition to increased body weight and adiposity, most of the other aforementioned phenotypes in VOK mice were also recapitulated by Cre virus–induced OGT deletion in VMH neurons. When body weight–matched virus-injected mice were subject to metabolic cage studies, we found that Cre virus–injected mice showed comparable food intake to GFP virus–injected mice (Fig. 6A). However, Cre virus–injected mice showed reduced energy expenditure with comparable physical activity, rectal temperature, and cold tolerance (Fig. 6, B and C, and fig. S1, C to E) relative to GFP virus–injected mice. They also exhibited higher RER, suggesting that there was an increased lipogenesis in Cre virus–injected mice (Fig. 6D).

In terms of the maladaptation of white adipose tissues, we found that Cre virus–injected mice showed adipocyte hypertrophy in different depots of white adipose tissues (Fig. 6, E and F). They also showed reduced lipolysis, indicated by the lower levels of serum nonesterified fatty acids after 15 hours of fasting compared to GFP virus–injected mice (Fig. 6G). Expression of phosphorylated and total adipose triglyceride lipase (ATGL) and HSL was reduced in scWAT and pgWAT of Cre virus–injected mice compared to GFP virus–injected mice (Fig. 6, H and I). The activity of the upstream kinase PKA was also down-regulated in white adipose tissues of Cre virus–injected mice (Fig. 6, J and K). Together, the phenotypes of Cre virus–injected mice further confirm that loss of OGT in VMH neurons down-regulates PKA-ATGL-HSL–mediated lipolysis of white adipose tissues.

**Fig. 7.**
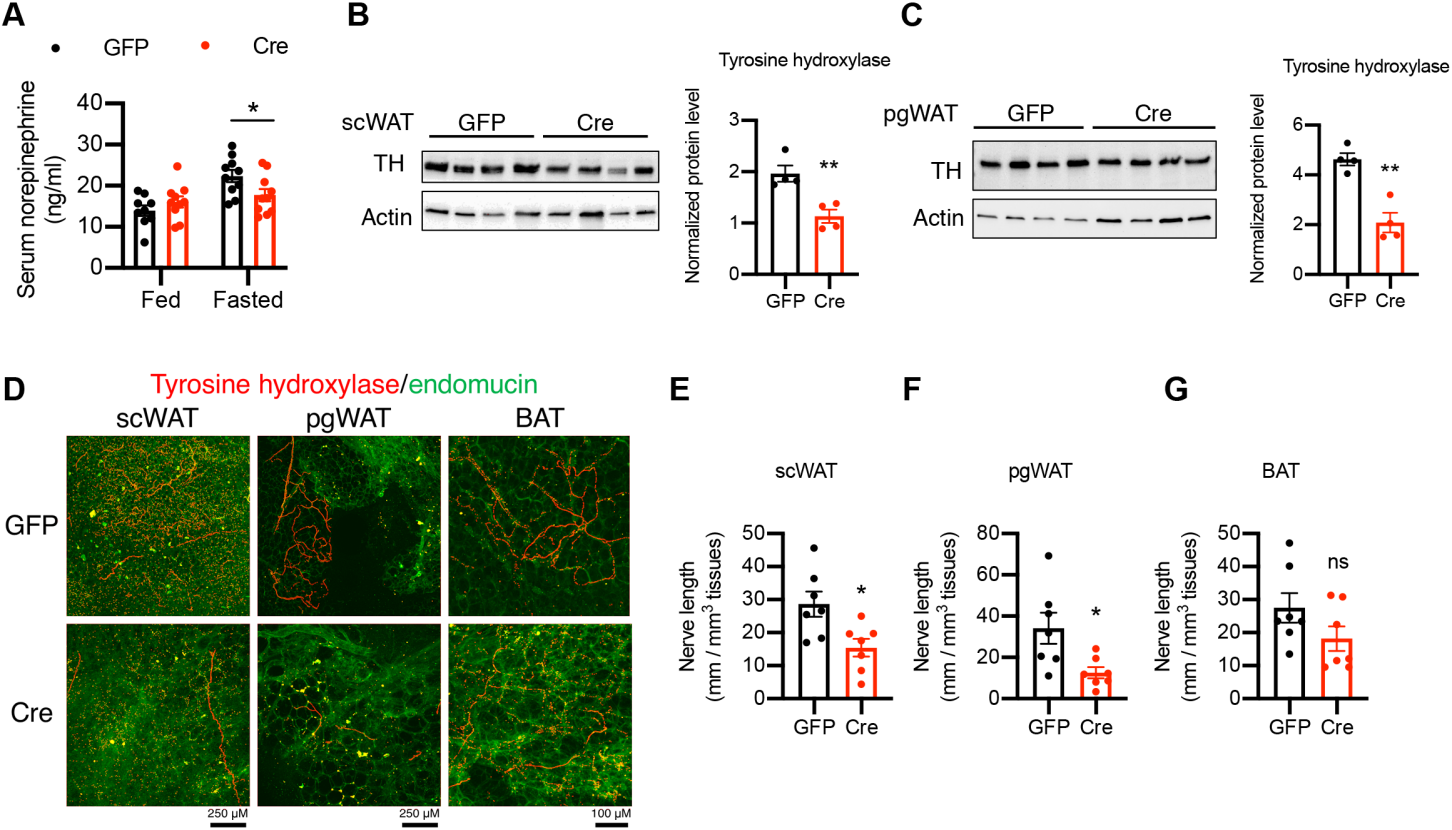
Mice with acute deletion of OGT in VMH neurons show reduced sympathetic activity and sympathetic innervations of white adipose tissues. (**A**) Serum norepinephrine levels of GFP and Cre mice fed ad libitum and after 15 hours of fasting. (**B** and **C**) Western blot of TH in scWAT (B) and pgWAT (C) of GFP and Cre mice after 6 hours of fasting. Densitometric quantification is shown on the right. (**D** to **G**) Representative immunostaining of TH (red) and endomucin (green) in scWAT, pgWAT, and BAT of GFP and Cre mice fed a normal chow diet ad libitum (D) and corresponding quantifications (E to G) from three GFP mice and four Cre mice 10 weeks after virus injection. GFP: *n* = 8 to 10 and Cre: *n* = 8 to 10 for serum NEFA and norepinephrine levels. Data are shown as means ± SEM. *P < 0.05 and **P < 0.01 by unpaired Student’s *t* test.

In agreement with the reduced activity of the sympathetic nervous system and impaired sympathetic innervation of white adipose tissues displayed by VOK mice, Cre virus–injected mice showed comparable serum norepinephrine levels fed ad libitum while lower serum norepinephrine levels after 15 hours of fasting (Fig. 7A). They also showed reduced levels of tyrosine hydroxylase in scWAT and pgWAT (Fig. 7, B and C), further confirming that OGT in VMH neurons is essential for the sympathetic outflow to white adipose tissues. Immunofluorescent staining of tyrosine hydroxylase and endomucin of adipose tissues showed that Cre virus–injected mice exhibited reduced sympathetic innervation of scWAT and pgWAT compared to GFP virus–injected mice, while the sympathetic innervation of BAT remained comparable (Fig. 7, D to G). Together, these results show that either prenatal or postnatal deletion of OGT in VMH neurons leads to reduced sympathetic activity and impaired sympathetic innervation of white adipose tissues.

### OGT controls the neuronal excitability of SF1 neurons

**Fig. 8.**
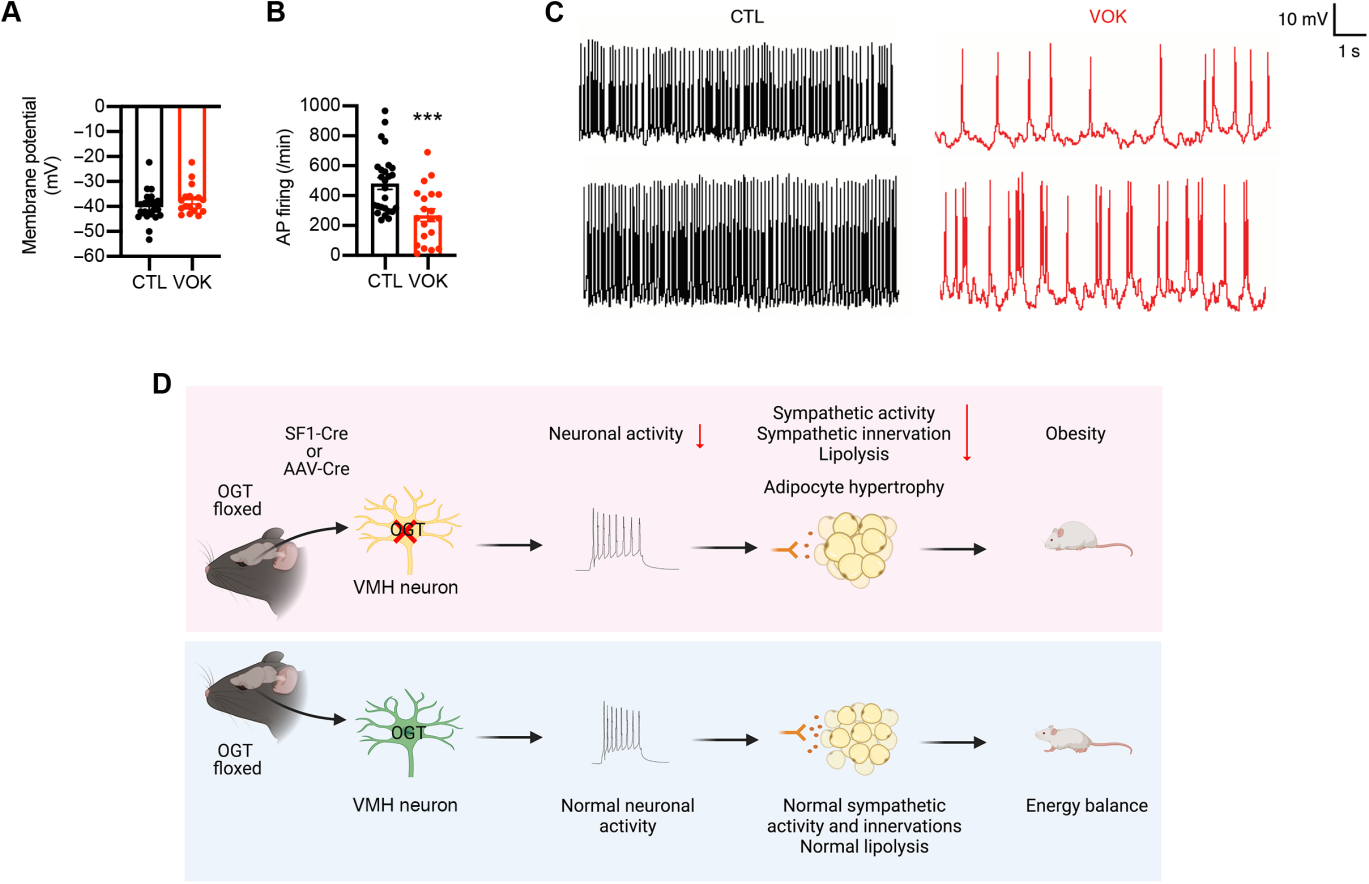
OGT is required for the neuronal activity of the VMH. (**A**) Basal membrane potential of SF1 neurons in CTL and VOK mice. (**B**) Firing rate of SF1 neurons in CTL and VOK mice. (**C**) Representative traces of action potentials of SF1 neurons in CTL and VOK mice. (**D**) The role of OGT in VMH neurons to regulate energy balance and white adipose tissue lipolysis. SF1 neurons from 5- to 7-week-old age-matched male CTL and VOK mice fed ad libitum on a normal chow diet were collected for electrophysiology. CTL SF1 neurons: *n* = 26 and VOK SF1 neurons: *n* = 20. Data are shown as means ± SEM. ***P < 0.001 by unpaired Student’s *t* test.

Given the phenotypes observed in both VOK and Cre virus–injected mice, we asked whether OGT is essential for VMH neuronal activity of the VMH. CTL and VOK mice with GFP-labeled SF1 neurons were generated to enable the identification of SF1 neurons during electrophysiological recordings. Whole-cell current clamp measurements demonstrated that the basal membrane potential of SF1 neurons was comparable between CTL and VOK neurons (Fig. 8A). However, SF1 neurons with OGT deletion showed a reduced spontaneous firing rate compared to CTL neurons (Fig. 8, B and C). The VMH has been considered an important site to regulate glucose homeostasis, with glucose-sensing neurons either activated or inhibited by glucose levels (*13*–*15*, *43*). When recording a collection of neurons’ responses to low-glucose treatment, we observed that VOK neurons showed a stronger excitatory response to low-glucose treatment (fig. S2, A and B). After quantifying the numbers of glucose-excited (GE) and glucose-inhibited (GI) neurons in the VMH of CTL and VOK mice, we observed that VOK mice showed a smaller number of GE neurons and a larger number of GI neurons (fig. S2, C and D). These results show that OGT deletion alters the glucose-sensing properties of SF1 neurons.

## DISCUSSION

Previous studies have greatly expanded our knowledge of the CNS control of metabolism, including energy balance and glucose homeostasis (*2*, *4*). It is of increasing interest to identify molecular players, signaling pathways, and neurocircuits involved as potential therapeutic targets to treat obesity and diabetes (*12*, *16*, *44*–*62*). Our findings have identified an antiobesogenic role of OGT in the VMH control of metabolism (Fig. 8D), as either chronic or acute deletion of OGT in VMH neurons reduces energy expenditure and induces obesity. OGT deletion in VMH neurons also reduces sympathetic activity and impairs sympathetic innervation of white adipose tissues, accounting for the reduced lipolysis and adipocyte hypertrophy. In addition, our findings have revealed that OGT is essential for the neuronal activity of VMH neurons, as deletion of OGT reduces action potential firing and alters the glucose-sensing properties of VMH neurons. Collectively, our findings reveal that OGT enables VMH neurons to promote fat mobilization of white adipose tissues and maintain energy balance, suggesting that OGT in VMH neurons is a set point of body weight control.

The VMH has long been recognized to control body weight by regulating food intake (*7*, *12*, *16*, *63*). In contrast, we have found that, without altering food intake, OGT deletion in VMH neurons rapidly exerts obesogenic effects by reducing energy expenditure (Figs. 2B; 3, A and C; 5D; and 6, A and C). It also reveals that VMH neurons rely on distinct pathways to regulate either food intake or energy expenditure to control body weight. In addition, VMH neurons have been widely considered thermogenic neurons as a large body of studies proposes that an intact genetic profile of VMH neurons is required to maintain energy balance when faced with the high-fat diet challenge, yet not under basal conditions (*12*, *45*, *46*, *64*). Our findings, however, show that OGT in VMH neurons plays an antiobesogenic role under basal conditions when mice are fed a normal chow diet (Figs. 2B and 5D), suggesting that OGT in the VMH acts as a set point of body weight control under basal conditions.

Previous studies have shown that the integrity of VMH neurons is required to maintain the sympathetic activity and outflow to peripheral tissues (*65*, *66*). Our data show that OGT deletion in VMH neurons reduces the activity of the sympathetic nervous system both systemically and locally in white adipose tissues (Figs. 4, D to F, and 7, A to C). In addition, we found that deletion of OGT in SF1 neurons remodels the sympathetic innervation of white adipose tissues, indicated by the lower density and length of the sympathetic nerves onto white adipose tissues (Figs. 4H and 7, D to G). The sympathetic innervation was also impaired when ventromedial hypothalamic OGT was acutely deleted in adult mice, suggesting that this phenotype was not due to developmental compensation and plasticity of peripheral tissue innervation. The reduced sympathetic activity and impaired sympathetic innervation of white adipose tissues further contribute to the deregulated lipolysis and fat accumulation (Figs. 3, E to H and 6, E to K). In light of our finding that OGT in VMH neurons is required for maintaining the sympathetic outflow to white adipose tissues, one future direction is to investigate how OGT in VMH neurons modulates the neurocircuits involving the autonomic nervous system that regulate sympathetic outflow to white adipose tissues.

O-GlcNAc signaling has long been proposed to serve as a nutrient sensor in multiple tissues, where glucose deprivation down-regulates cellular O-GlcNAcylation levels due to the lower availability of intracellular UDP-GlcNAc. On the other hand, *O*-GlcNAc signaling also serves as a stress sensor in some tissues, where a global increase in cellular O-GlcNAcylation levels is observed in conditions of nutrient deprivation (*18*). Here, we report that 24 hours of fasting is associated with increased OGT expression and O-GlcNAcylation level in the VMH, suggesting that OGT in the VMH serves as a stress sensor. Whether OGT in the VMH plays a nutrient-sensing role under other circumstances needs further investigation. Moreover, we propose an antiobesogenic and prolipolytic role of ventromedial hypothalamic OGT in controlling body weight, in contrast to the previously reported antilipolytic role of OGT in peripheral tissues (*27*), further strengthening the concept that O-GlcNAc signaling mediates nutritional and hormonal regulation of metabolism in a tissue-specific manner (*23*–*31*).

Previous studies of the role of OGT in the CNS suggest that OGT is essential for neuronal survival, as deletion of OGT in sensory neurons leads to axonal dieback and neuronal death (*67*). It has also been shown that OGT is necessary for excitatory synapse maturity or neuronal activity by modulating the voltage-dependent Kcnq3 channel (*32*, *68*). In our model, deletion of OGT in SF1 neurons does not alter neuron numbers or the gross cytoarchitecture of the VMH (fig. S1, A and B). Instead, we found that OGT deletion in SF1 neurons impairs neuronal activity by reducing spontaneous action potential firing (Fig. 8, A to C). It also alters the glucose-sensing properties of SF1 neurons (fig. S2, A to D).

One of the limitations of our study is that we did not further examine how VMH O-GlcNAcylation affects systematic metabolism in a sex-specific manner. Previous publications have shown that manipulation of gene expression in the VMH affects male and female mice differently. For example, Xu *et al*. (*69*) showed that estrogen receptor-a deletion in SF1 neurons led to obesity and higher adiposity specifically in female mice. Cheung *et al*. (*70*) showed that Vglut2 knockdown in the SF1 neurons led to less weight gain on high-fat diet only in female mice while both sexes were affected in their anxiety levels. Fagan *et al*. (*55*) also showed that the deletion of metabotropic glutamate receptor subtype 5 only impaired glucose and lipid metabolism in female mice. Further investigation is needed to elucidate whether female mice are affected by OGT deletion in SF1 neurons.

In summary, the present study furthers the knowledge of how VMH neurons regulate sympathetic innervation and lipid metabolism. Our study reveals an antiobesogenic role of OGT in the neural control of metabolism and suggests central OGT as a potential therapeutic target to combat obesity.

## MATERIALS AND METHODS

### Mice

*Ogt*-floxed mice on C57BL/6J background were provided by S. Jones (University of Louisville). *Sf1-Cre* mice were provided by S. Diano at Yale University and purchased from the Jackson Laboratory. *Sf1-Cre-GFP* mice were provided by S. Diano at Yale University and have been maintained on a mixed background. To delete OGT selectively in SF1 neurons, *Ogt*-floxed mice were crossed with *Sf1-Cre* mice to generate *Sf1-Cre; Ogt^f/Y^* and *Sf1-Cre; Ogt^f/f^* mice and their wild-type littermates. To generate mice with GFP-labeled SF1 neurons, *Ogt*-floxed mice were crossed with *Sf1-Cre-GFP* mice. All mice were kept on a 12-hour light/12-hour dark cycle. All mice had free access to water and a standard chow diet or 60% high-fat diet (Research Diets). The sex and age of the mice used in the experiment were specified in the text. All procedures have been approved by the Institutional Animal Care and Use Committee of Yale University.

### Metabolic assays

Body weights were recorded each week after weaning. Body composition was determined with the EchoMRI system. Metabolic cage assays were performed with the Promethion multiplexed metabolic measurement system and TSE system. To acclimate to the metabolic cage environment, mice were first single-housed for 5 days and then moved to the metabolic cages for another 3 days before the experiments. Data were collected consecutively for 7 days, and only the data from the last 4 days were used to determine gas exchange, food intake, and ambulatory activity of the mice. Heat production was calculated and adjusted to body weight (*71*). Body temperature was measured rectally using a thermos-coupler (Physitemp). For glucose, insulin, or 2DG tolerance tests, 15-hour-fasted mice were injected intraperitoneally with glucose (1.5 g/kg of body weight), 6-hour-fasted mice were injected intraperitoneally with insulin (1 U/kg of body weight), and 4-hour-fasted mice were injected intraperitoneally with 2DG (200 mg/kg of body weight; 2DG was purchased from Sigma-Aldrich). Blood glucose from tail-vein blood collected at the designated times was measured using a Nova Max glucometer.

### Electrophysiology

Mice were anesthetized with isoflurane and euthanized by decapitation. The brain was gently removed from the skull and chilled in 4°C oxygenated high-sucrose solution containing the following: 220 mM sucrose, 2.5 mM KCl, 1.23 mM NaH_2_PO_4_, 26 mM NaHCO_3_, 1 mM CaCl_2_, 6 mM MgCl_2_, and 10 mM glucose (pH 7.3) with NaOH. The brain was trimmed to a large block containing the hypothalamus and then sliced on a vibratome. Coronal, 300-μm slices were cut through the full extent of the VMH. Slices were maintained in artificial cerebrospinal fluid (aCSF; containing 126 mM NaCl, 2.5 mM KCl, 1.2 mM MgCl_2_, 2 mM CaCl_2_ × 2H_2_O, 1.2 mM NaH_2_PO_4_ × H_2_O, 26 mM NaHCO_3_, and 10 mM glucose) for 1 hour at room temperature in 95% O_2_ and 5% CO_2_ saturated aCSF before recordings. Perforated whole-cell current clamp was used to observe membrane potential and spontaneous action potential firings. All data were sampled at 3 to 10 kHz and filtered at 1 to 3 kHz. The patch pipette was made of borosilicate glass with a Sutter puller. The tip resistance of the recording pipettes was 2 to 4 megohms after filling with a pipette solution containing the following: 135 mM potassium methanesulfonate, 2 mM MgCl_2_, 10 mM Hepes, 1.1 mM EGTA, 2 mM Mg–adenosine triphosphate, 10 mM Na_2_-phosphocreatine, and 0.3 mM Na_2_-GTP (pH 7.3) with KOH. All data were sampled and analyzed with AxoGraph X.

### Stereotaxic virus injection

The AAV8/hSyn-eGFP or AAV8/hSyn-Cre-GFP virus (University of North Carolina VectorCore) was injected bilaterally into the VMH as previously described. We anesthetized 12-week-old *Ogt*-floxed male mice with bupivacaine [8 mg/kg, intraperitoneally (i.p.)], ketamine (100 mg/kg), xylazine (10 mg/kg, i.p.), and Buprenex (0.05 mg/kg, subcutaneously) and placed them in stereotaxic apparatus. A pulled glass needle was inserted into the VMH (from bregma, anteroposterior: −1.4 mm; mediolateral: 0.42 mm; and dorsoventral: −5.9 mm). We administered 0.4 μl of AAV virus (3.0 × 10^12^ viral genomes/ml) into one side of the VMH. At 10 min following the injection to allow for adequate dispersal and absorption of the virus, the same volume of the virus was injected into the contralateral VMH. Animals were administered with Metacam (1 mg/kg, i.p.) for three consecutive days after the surgery and were given a 2-week recovery before metabolic analysis was performed.

### Western blot

Anti-OGT (24083), anti-HSL (4107), anti–phospho-HSL S563 (4139), anti–phospho-HSL S565 (4137), anti-ATGL (2138S), anti–phospho-PKA substrate (100G7E), anti–tyrosine hydroxylase (2792), anti–acetyl–coenzyme A (CoA) carboxylase (3662), and anti–phospho-acetyl-CoA carboxylase S79 (3661) were purchased from Cell Signaling Technology. Anti-RL2 (ab2739) and anti–phospho-ATGL S406 (ab135093) were purchased from Abcam. Anti-NeuN (MAB377) and anti–tyrosine hydroxylase (Ab152) were purchased from Millipore Sigma. Anti-OGT (85370) and anti-actin (1170) were purchased from GenuIN BIOTECH. Anti-actin (sc-8432) was purchased from Santa Cruz Biotechnology. Anti-endomucin (AF4666) was purchased from R&D Systems. Tissues were homogenized and lysed in buffer containing 1% NP-40, 150 mM NaCl, 0.1 mM EDTA, 50 mM tris-HCl, proteinase inhibitors, and protein phosphatase inhibitors. The lysates were then washed and boiled in SDS loading buffer. Equal amounts of protein lysates were resolved on SDS–polyacrylamide gel electrophoresis gels and transferred to polyvinylidene difluoride membrane. The membranes were blocked in 5% bovine serum albumin (BSA) and incubated with various primary antibodies overnight at 4°C. After three washes, the membranes were incubated with peroxidase-conjugated secondary antibodies for 1 hour and visualized with enhanced chemiluminescence substrate. The Bio-Rad ChemiDoc system was used to visualize the blots.

### Histology

Mice were fasted for 6 hours before sacrifice. Tissue samples were dissected, fixed in 4% paraformaldehyde for 48 hours, and embedded in paraffin blocks. H&E stains were performed by the Histology Core in the Department of Comparative Medicine. For immunofluorescent staining, mice were anesthetized for intracardial perfusion of PBS, followed by 4% paraformaldehyde with 0.08% glutaraldehyde. Brain and adipose depots were dissected and postfixed in 4% paraformaldehyde overnight. Coronal brain sections (50 μm) were prepared using a vibratome. Tissue sections were blocked with 3% BSA and 0.2% Triton X-100 in 0.1 M phosphate-buffered saline (PBS) and were incubated with primary antibodies (1:100 dilution; CST, anti-OGT, 24083; Abcam, anti-RL2, ab2739) overnight and secondary antibodies [1:400; Alexa Fluor 488 anti-rabbit immunoglobulin G (IgG), Alexa Fluor 594 anti-rabbit IgG, Alexa Fluor 594 anti-mouse IgG, and Alexa Fluor 647 anti-rabbit IgG] for 1 hour. Immunofluorescent staining and clearance of tissues were performed following previous literature (*72*). The slides were mounted with VECTASHIELD antifade mounting medium (Vector Laboratories) and saved at 4°C until imaging. A Keyence digital microscope and the Leica SP5 confocal system were used for H&E stain imaging and fluorescent imaging.

### Enzyme-linked immunosorbent assay

Fresh serum samples and homogenized frozen tissues were used for norepinephrine (Abnova, KA3836 and KA1891) and glucagon (Crystal Chem, #81518) enzyme-linked immunosorbent assays. Assays were performed according to the manufacturer’s protocols.

### Statistical analysis

Results are presented as means ± SEM. The comparisons were carried out using a two-tailed unpaired Student’s *t* test or one-way analysis of variance (ANOVA) followed by Tukey-adjusted multiple comparisons. Data were plotted with GraphPad Prism. Statistical tests used are stated in the figure legends. Materials and Methods should provide sufficient information to allow replication of the results. For the blinding to electrophysiology experiments, CTL or VOK mice were named after neutral codes and then given to an investigator not involved in the design of the experiments to perform treatment and electrophysiological recording. For the randomization of the stereotaxic virus injection, mice in the same cohort and with comparable body weights were randomly selected to receive the GFP- or Cre-expressing virus. During the experiments, all eligible mice in the same cohort were included in the measurements unless they were diagnosed with tumors, malocclusion, or other diseases by Yale Veterinary Clinical Services during the project.

## Notes

View/request a protocol for this paper from *Bio-protocol*.
