# Supplementary material for: Ventromedial hypothalamic OGT drives adipose tissue lipolysis and curbs obesity
Source: Sci Adv. 2022 Aug 31;8(35):eabn8092. doi: 10.1126/sciadv.abn8092 (PMC9432828; doi:10.1126/sciadv.abn8092)
Supplement: Supplementary file 1 — Figs. S1 and S2 [file sciadv.abn8092_sm.pdf]

Science Advances
AAAS

Supplementary Materials for

Ventromedial hypothalamic OGT drives adipose tissue lipolysis and curbs obesity

Qi Wang *et al.*

Corresponding author: Xiaoyong Yang, xiaoyong.yang@yale.edu

Sci. Adv. **8**, eabn8092 (2022)
DOI: 10.1126/sciadv.abn8092

This PDF file includes:

Figs. S1 and S2

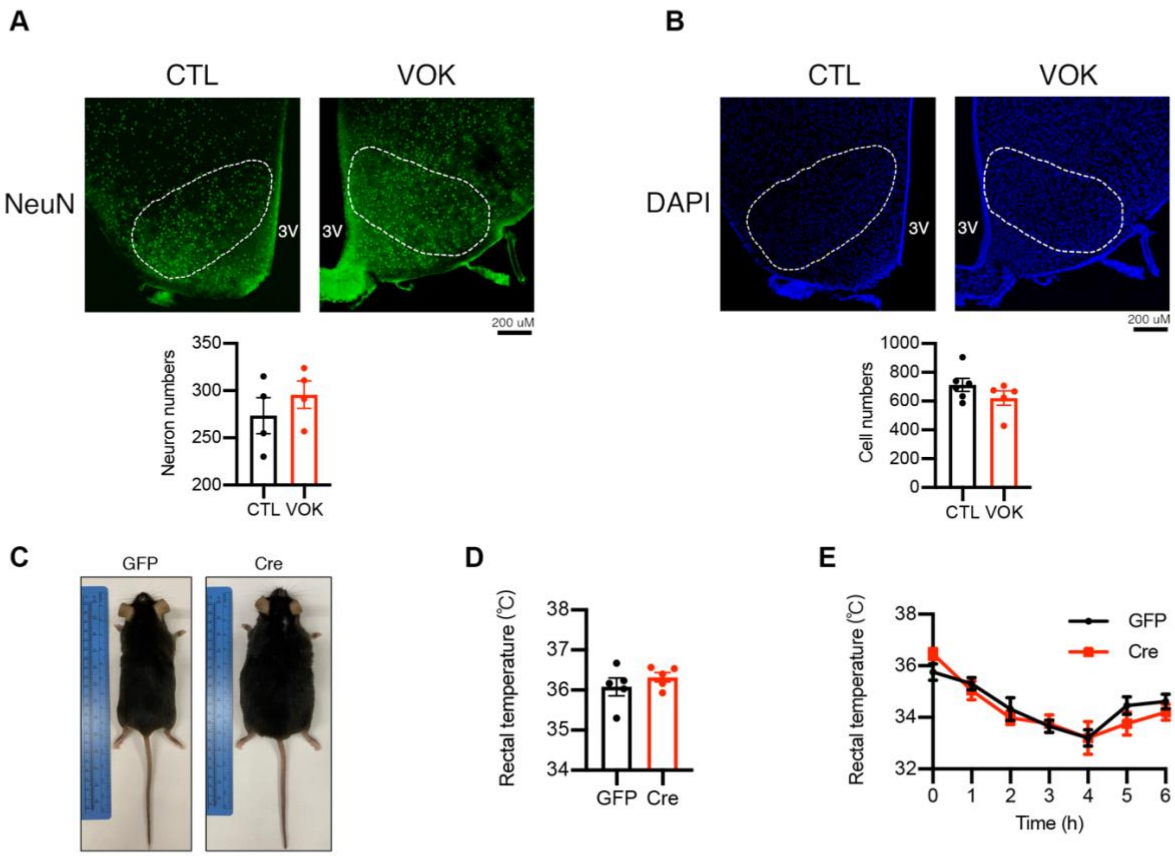

Fig. S1. Chronic deletion of OGT in SF1 neurons does not alter the gross cytoarchitecture of the VMH. Acute deletion of OGT in VMH neurons leads to obesity in mice fed on a normal chow diet with intact thermogenesis and counter-regulatory response.

(A-B) Immunostaining of NeuN (A) and DAPI (B) in the VMH of CTL and VOK mice showing unaltered cytoarchitecture of the VMH after OGT deletion. Quantifications of neuron numbers and cell numbers are shown on the bottom. Scale bar: 200 um. Data are shown as mean ± SEM by unpaired Student's *t*-test.

(C) Representative images of GFP and Cre mice 32 weeks after virus injection.

(D) Rectal temperature of GFP and Cre mice under room temperature.

(E) Rectal temperature of GFP and Cre during 4℃ acute cold challenge. GFP: n=5, Cre: n=5 for rectal temperature measurement.

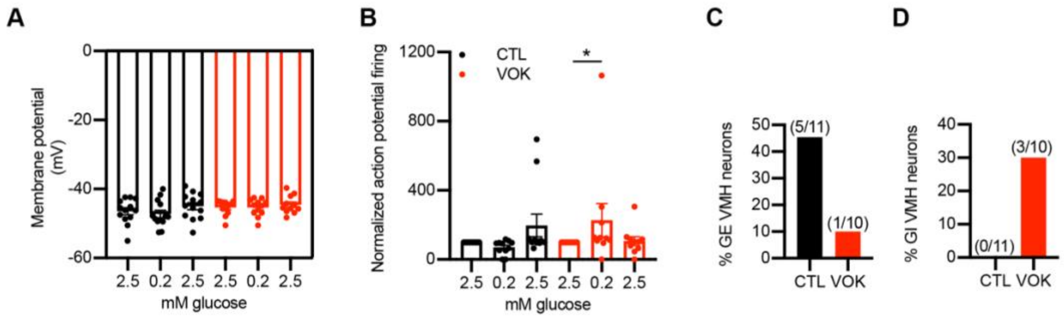

Fig. S2. Deletion of OGT in SF1 neurons affects glucose sensing.

(A) Membrane potential of SF1 neurons in CTL and VOK mice during glucose treatment.
(B) Normalized action firing rate of SF1 neurons in CTL and VOK mice during glucose treatment.
(C-D) Percentages of GE (C) and GI (D) neurons in the VMH of CTL and VOK mice. SF1 neurons from age-matched male CTL and VOK mice aging 5-6 weeks fed *ad libitum* on a normal chow diet were collected for electrophysiology. CTL SF1 neurons: n=11, VOK SF1 neurons: n=10. Data are shown as mean ± SEM. * $p < 0.05$ by unpaired Student's *t*-test. GE, glucose-excited; GI, glucose-inhibited.
